# Response and Toxicity to the Second Course of 3 Cycles of ^177^Lu-PSMA Therapy Every 4 Weeks in Patients with Metastatic Castration-Resistant Prostate Cancer

**DOI:** 10.3390/cancers13102489

**Published:** 2021-05-20

**Authors:** Sazan Rasul, Tim Wollenweber, Lucia Zisser, Elisabeth Kretschmer-Chott, Bernhard Grubmüller, Gero Kramer, Shahrokh F. Shariat, Harald Eidherr, Markus Mitterhauser, Chrysoula Vraka, Werner Langsteger, Marcus Hacker, Alexander R. Haug

**Affiliations:** 1 Division of Nuclear Medicine, Department of Biomedical Imaging and Image-guided Therapy, Medical University of Vienna, 1090 Vienna, Austria; sazan.rasul@meduniwien.ac.at (S.R.); tim.wollenweber@meduniwien.ac.at (T.W.); lucia.zisser@meduniwien.ac.at (L.Z.); elisabeth.kretschmer-chott@meduniwien.ac.at (E.K.-C.); harald.eidherr@meduniwien.ac.at (H.E.); markus.mitterhauser@meduniwien.ac.at (M.M.); chrysoula.vraka@meduniwien.ac.at (C.V.); werner.langsteger@meduniwien.ac.at (W.L.); marcus.hacker@meduniwien.ac.at (M.H.); 2 Department of Urology, Medical University of Vienna, 1090 Vienna, Austria; bernhard.grubmueller@meduniwien.ac.at (B.G.); gero.kramer@meduniwien.ac.at (G.K.); shahrokh.shariat@meduniwien.ac.at (S.F.S.); 3 Department of Urology, Weill Cornell Medical College, New York, NY 10065, USA; 4 Department of Urology, Second Faculty of Medicine, Charles University, 15006 Prague, Czech Republic; 5 Institute for Urology and Reproductive Health, I.M. Sechenov First Moscow State Medical University, 119991 Moscow, Russia; 6 Department of Urology, University of Texas Southwestern Medical Center, Dallas, TX 75390, USA; 7 Ludwig Boltzmann Institute Applied Diagnostics, 1090 Vienna, Austria; 8 Christian Doppler Laboratory for Applied Metabolomics (CDL AM), Medical University of Vienna, 1090 Vienna, Austria

**Keywords:** PSMA-RLT, ^177^Lu-PSMA, PSA, mCRPC, prostate cancer

## Abstract

**Simple Summary:**

The [^177^Lu]Lu-PSMA radioligand therapy (PSMA-RLT) has emerged as a successful treatment option in patients with metastatic castration-resistant prostate cancer (mCRPC). Nevertheless, the therapeutic protocol of this treatment is still heterogeneous in many centers, in terms of the number of cycles and the interval between the cycles. Recently, we published the clinical impact of a homogeneous PSMA-RLT protocol that has been applied in our clinic since we started offering this treatment to patients with mCRPC. The outcomes were supportive and promising for analyzing the efficacy and toxicity of using the same treatment regimen in patients who benefited from the first treatment course. Based on the results, we concluded that a second course of three cycles of standardized PSMA-RLT with only a 4-week interval between the cycles is safe and offers favorable tolerability, response rates, overall survival, and progression-free survival, rendering it a promising alternative for the retreatment of mCRPC patients who have formerly responded well to PSMA-RLT.

**Abstract:**

Background: We investigated the response rate and degree of toxicity of a second course of three cycles of [^177^Lu]Lu-PSMA radioligand therapy (PSMA-RLT) every 4 weeks in mCRPC patients. Methods: Forty-three men (71.5 ± 6.6 years, median PSA 40.8 (0.87–1358 µg/L)) were studied. The response was based on the PSA level 4 weeks after the third cycle. The laboratory parameters before and one month after the last cycle were compared. Kaplan–Meier methods were used to estimate the progression-free survival (PFS) and overall survival (OS), and the Cox regression model was performed to find predictors of survival. Results: Twenty-six patients (60.5%) exhibited a PSA reduction (median PSA declined from 40.8 to 20.2, range 0.6–1926 µg/L, *p* = 0.002); 18 (42%) and 8 (19%) patients showed a PSA decline of ≥50% and ≥80%, respectively. The median OS and PFS were 136 and 31 weeks, respectively. The patients with only lymph node metastases survived longer (*p* = 0.02), whereas the patients with bone metastases had a shorter survival (*p* = 0.03). In the multivariate analysis, only the levels of PSA prior to the therapy remained significant for OS (*p* < 0.05, hazard ratio 2.43, 95% CI 1.01–5.87). The levels of hemoglobin (11.5 ± 1.7 g/dL vs. 11 ± 1.6 g/dL, *p* = 0.006) and platelets (208 ± 63 g/L vs. 185 ± 63 g/L, *p* = 0.002) significantly decreased one month after cycle three, though only two grade 3 anemia and one grade 3 thrombocytopenia were recorded. Conclusion: A further intensive PSMA-RLT course is well tolerated in mCRPC patients and associated with promising response rates and OS.

## 1. Introduction

Prostate cancer is one of the most common cancers and one of the leading oncologic causes of death in men in western countries. In these patients, particularly in those with aggressive, metastatic or castration-resistant prostate cancer (mCRPC), the levels of prostate-specific membrane antigen, also called glutamate carboxypeptidase type II and abbreviated as PSMA, are elevated up to 1000 times the normal value and are inversely correlated with the levels of androgens [1]. These receptors are a highly potent target in the diagnosis and treatment of patients with prostate tumors. Therefore, radionuclides targeting these peptides, such as [^68^Ga]Ga-PSMA-11 ligand positron emission tomography (^68^Ga-PSMA PET), which is widely applied as a non-invasive molecular method for imaging prostate cancer, and [^177^Lu]Lu-PSMA-617 radio-ligand therapy (PSMA-RLT), which has emerged as a valuable treatment in patients with mCRPC, are currently available. Although this novel therapy has not yet been approved for clinical use, it has been successfully administered to patients with mCRPC, based on the results of numerous studies [2,3,4,5]. Despite encouraging favorable outcomes of these trials on the efficacy and safety of PSMA-RLT [2,6], this therapy is presently used in mCRPC patients only as a last therapy option when other available standard medical procedures have failed to show clinical improvement. The therapeutic protocol in many centers, however, is quite heterogenous with treatments differing between two to six cycles of 3.7–9.3 GBq PSMA-RLT every 6 to 8 weeks [7]. Most recently, the TheraP study, a multicenter, unblinded, randomized phase 2 trial involving 11 centers in Australia, demonstrated a more frequent PSA response in mCRPC men treated with PSMA-RLT than in patients receiving cabazitaxel at the same stage of the disease. In addition, the results of this study reported fewer serious adverse events in men treated with PSMA-RLT than with cabazitaxel [8]. 

We lately published the clinical impact of a homogeneous PSMA-RLT protocol consisting of three cycles of 7400 MBq PSMA-RLT with 4-week intervals, which has been used in our clinic since we started offering this treatment to patients with mCRPC [2]. The results of this standardized treatment protocol were very favorable concerning the rate of response, overall survival (OS) as well as progression-free survival (PFS) and therapy-related toxicity, also in comparison to the findings of previous studies [4,6,9,10]. These observations and outcomes were highly supportive to analyze the efficacy and toxicity of applying the same treatment regimen in patients who gained benefit from the first treatment course. Hence, in this study, we aimed to elucidate the response rate and toxicity in mCRPC patients who underwent a second course consisting of three cycles of highly standardized PSMA-RLT every 4 weeks.

## 2. Patients and Methods

### 2.1. Patients

This retrospective study included data from all mCRPC patients being referred to the Department of Nuclear Medicine, Medical University of Vienna, Vienna General Hospital, between September 2015 and May 2020 who were eligible and scheduled for the second course of PSMA-RLT. The median distance between the 1st and the 2nd therapy course was 16 weeks (range 4–96 weeks) and the median duration of follow-up of these patients was 30 months (ranged 4–50). In an interdisciplinary tumor board, the initiation of PSMA-RLT was endorsed for all the mCRPC patients studied. The decisive requirements for applying a second course of treatment were a sufficient response rate, high tumor burden, and good tolerability of the first PSMA-RLT course in the absence of clinical and laboratory signs of severe therapy toxicities among the treated patients. Furthermore, the presence of PSMA-positive lesions in a ^68^Ga-PSMA PET scan conducted for each patient before the start of the 2nd treatment course was mandatory for receiving the therapy. The protocol of the performed ^68^Ga-PSMA PET/computed tomography (CT) or PET/magnetic resonance imaging (MRI) scan for these patients was previously described in the study by Grubmüller et al. [11]. 

### 2.2. Medical Care of the Patients and the Applied PSMA-RLT Protocol

As described previously [2,12], each therapy course routinely applied in our clinic was composed of 3 cycles of PSMA-RLT acquired from ABX GmbH (Radeberg, Germany) with 4-week intervals between every cycle. For each of them, every patient was hospitalized, received medical care and was monitored for at least 72 h. Their Karnofsky performance score, and Eastern Cooperative Oncology Group (ECOG) index were then determined by an experienced medical doctor. Laboratory parameters such as complete blood count, biochemistry, and PSA levels were assessed for each patient at each this visit and 1 month after the last 3rd cycle. Based on the laboratory results, the common terminology criteria for adverse events (CTCAE), version 4.0, were considered to evaluate treatment toxicities. The therapy was intravenously administered following paragraph § 8 of the Austrian Medicinal Products Act (AMG). Thirty minutes prior to the application of the PSMA-RL, every patient obtained 1 liter of normal saline infusion at 300 mL/h. Subsequently, in order to enable imaging assessments of treatment responses, all patients received a second [^68^Ga]Ga-PSMA-11 whole-body PET scan 4–6 weeks after the last cycle of the therapy.

### 2.3. Statistical Analysis

All statistical methods mentioned in this study were performed using IBM SPSS Statistics for Windows, version 24.0 (IBM Corp., Armonk, NY, USA). The Kolmogorov–Smirnov test was conducted to estimate the distribution of all data used in this study. Normally distributed data were shown as mean ± standard deviation, whereas non-normally distributed data were displayed as median and range, and log-10 transformed for analysis. All categorical variables were presented in percentages and number of recorded cases, and the comparison of laboratory parameters before and 1 month after acquiring the last 3rd cycle of the PSMA-RLT was conducted using the paired *t*-test. In all patients, PFS and OS were estimated using Kaplan–Meier estimates and a Cox proportional hazard model. Additionally, log-rank analyses (Mantel–Cox test) were performed to examine the impact of factors such as type and location of metastasis and history of previously receiving other therapies like hormonal as well as chemo- and Ra-223 (Xofigo^®^) therapy before the start of PSMA RLT on the survival and PFS in this studied cohort. The PFS was defined as the time from the first cycle of the second course of therapy until the detection of PSA progression. OS was ascertained from the date of the first cycle of the first course of therapy as well as from the date of the first cycle of the second course of therapy until the date of death or until the date of the last hospital follow-up. For all results, a *p*-value < 0.05 was considered statistically significant.

## 3. Results

Collectively, 43 mCRPC patients (aged 71.4 ± 6.6 years) were valid to acquire the second course of PSMA-RLT, which was composed of three cycles of standardized [^177^Lu]Lu-PSMA-617 (7351 ± 647 MBq) every 4 weeks. The clinical characteristics of these patients prior receiving the second PSMA-RLT therapy are presented in Table 1. Among this cohort, 26 patients (60.5%) responded to the first PSMA-RLT course with a PSA reduction of more than 50%. The Karnofsky score was lower than 80% in only 16 (37.2%) patients, and equal and higher than 80% in 27 (62.8%) patients. The ECOG index was 0 in 8 (18.6%), 1 in 26 (60.5%) and 2 in 9 (20.9%) patients. Twenty-seven (62.8%) patients had a history of enzalutamide or abiraterone therapy, while 30 (69.7%) patients were previously treated with chemotherapy (docetaxel and/or cabazitaxel) and only 12 (27.9%) patients were treated with Ra-223 (Xofigo^®^). Between the first and second therapy courses, the patients did not obtain newly initiated treatments with chemo- and Ra-223 therapies. However, in the men already treated with abiraterone or enzalutamide, these therapies were continued in individual patients between the two PSMA-RLT courses without starting new antiandrogenic therapies. 

The distributions of metastatic lesions on the basis of the [^68^Ga]Ga-PSMA-11 scan were as follows: lymph node only (M1a) in 8 (18.6%), bone ± lymph node without visceral metastasis (M1b) in 27 (62.8%), and any visceral metastasis (M1c) in 8 (18.6%) patients, all shown in Table 1. 

### 3.1. Response Rate and Clinical Effects of Second PSMA-RLT Course

**Figure 1:**
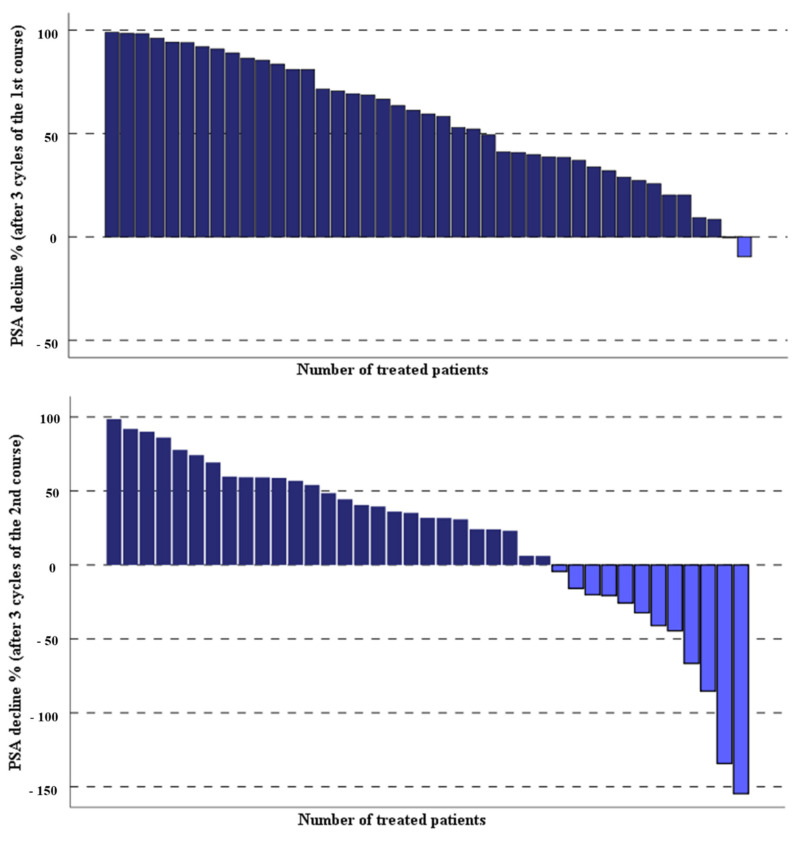
Percentage of PSA change in the studied patients after two courses of the highly standardized PSMA-RLT, each three cycles with 4-week interval.

In Table 2, the laboratory parameters of the entire studied mCRPC patients before and 1 month after the third last cycle of the second course of PSMA-RLT applied every 4 weeks have been compared. The PSA levels of the treated patients decreased significantly after three cycles of PSMA-RLT compared with baseline, median PSA 40.8 (range 0.87–1358 µg/L) vs. 20.2 (range 0.6–1926 µg/L), *p* = 0.002. Overall, 26 out of 43 (60.5%) patients demonstrated any decrease in PSA levels, 18 out of 43 (42%) had a PSA decline of ≥50%, and 8 of 43 (19%) patients showed a PSA decrease of ≥80%. The percentage of the PSA decline after both treatment courses of highly standardized PSMA-RLT, each three cycles with a 4-week interval, in all patients studied are depicted in Figure 1. 

Moreover, levels of hemoglobin (Hb) (11.5 ± 1.7 g/dL vs. 11 ± 1.6 g/dL, *p* = 0.006) and platelets (208 ± 63 g/L vs. 185 ± 63 g/L, *p* = 0.002) one month after the third cycle were significantly lower compared to the baseline (Table 2). However, only two cases of grade 3 anemia and one case of grade 3 thrombocytopenia were observed among all the treated patients (Table 3). In addition, no statistically significant changes in the levels of leukocyte, creatinine, alkaline phosphatase, and lactate dehydrogenase were observed when we compared their basal values with those one month after treatment with three cycles of PSMA-RLT (Table 2). No patients with severe gastrointestinal adverse events, as well as no patients with acute parotitis and myelodysplastic syndrome, were reported during the second PSMA-RLT course. 

### 3.2. Overall Survival of Patients Treated with Second PSMA-RLT Course

**Figure 2:**
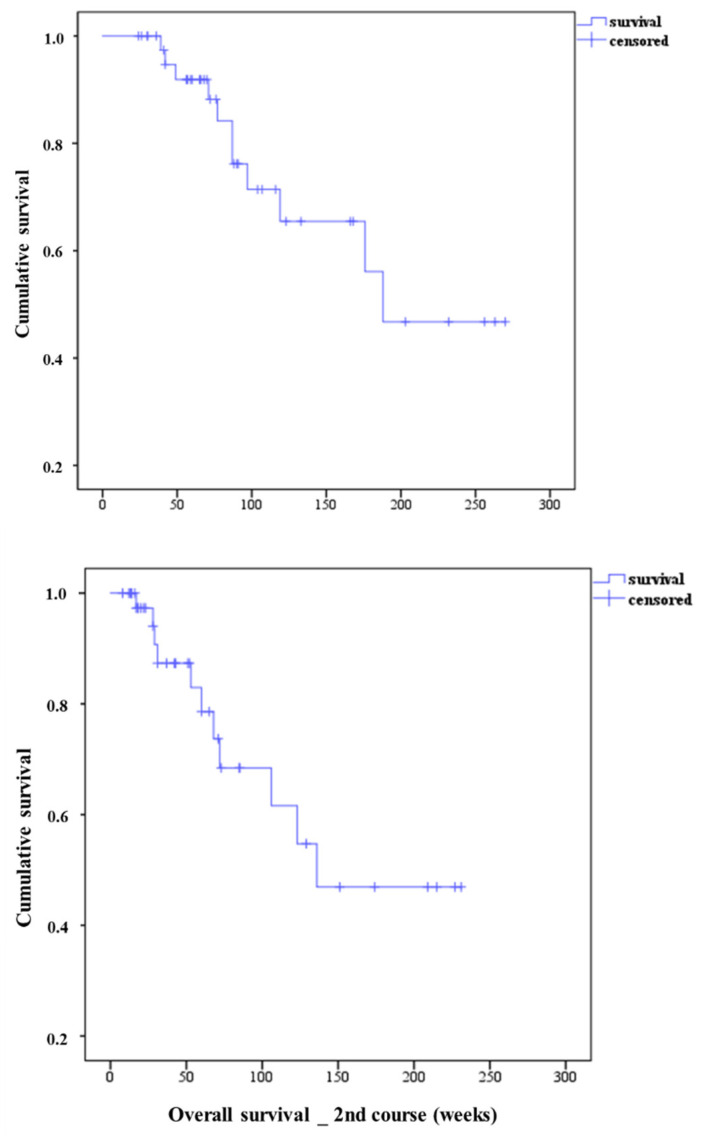
Kaplan–Meier plots of the treated patients received collectively two courses of PSMA-RLT, each course composed of three cycles of 7400 MBq PSMA-RLT every 4 weeks. The median overall survival from the time of the first cycle of the first PSMA-RTL course was 188 weeks, whereas the median survival from the time of the first cycle of the second PSMA-RLT course was 136 weeks.

Kaplan–Meier plots of the entire treated patients revealed a median OS of 188 weeks from the beginning of the first cycle of the first course, and a median OS of 136 weeks from the start of the first cycle of the second course of the treatment, shown in Figure 2. Among the collective populations studied, the median PFS from the time of the beginning of the second therapy course was 31 weeks (95% CI 26–36), while from the first cycle of the first course to the PSA progression was 27 weeks (95% CI 22–32). In Table 4, we have presented the OS as well as the PFS calculated from the time of the first cycles of both PSMA-RLT courses depending on the type of metastases present in the treated patients. As shown in that table, after receiving the first and second therapy courses, the shortest OS was observed among the patients with M1c, whereas the shortest PFS was seen in the patients with M1b. 

**Figure 3:**
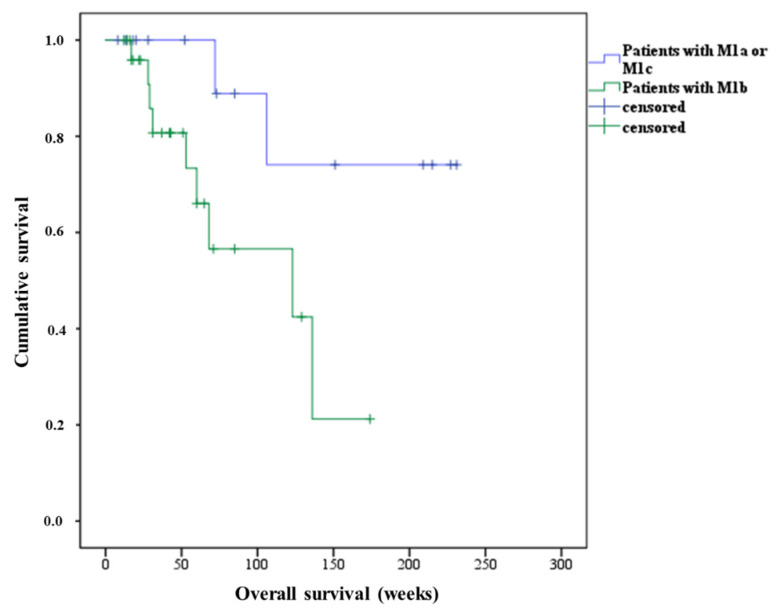
Kaplan–Meier plots using log-rank (Mantel–Cox) test illustrate overall survival of mCRPC patients treated with a second course of three cycles of PSMA-RLT. Male patients with bone metastasis (M1b) had significantly shorter survival compared to patients with lymph node (M1a) or visceral metastases (M1c), median survival 123 weeks vs. not reached, *p* = 0.03, 95% CI 2.42–243.

**Figure 4:**
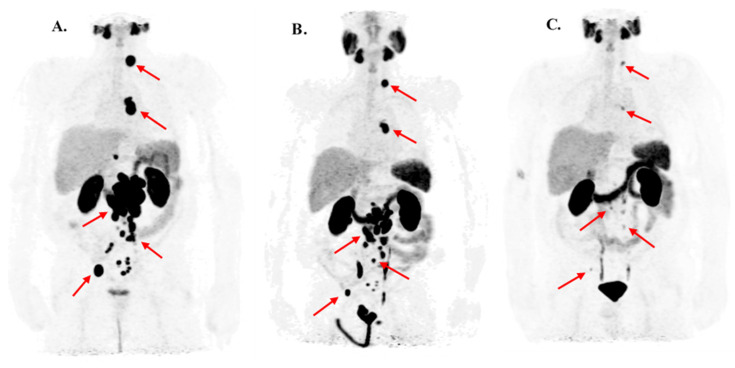
The [68Ga]-Ga-PSMA PET examination of an mCRPC patient with only lymph node metastasis who received two courses (each three cycles with 4-week interval) of PSMA-RLT. The [^68^Ga]GaPSMA-11 PET images of a 76-year-old mCRPC patient. (**A**): prior the first cycle of first course of PSMA-RLT with clearly PSMA-overexpressed lymph node (LN) metastases in upper and lower diaphragm and a PSA value of 597 ng/mL. (**B**): one month after the third cycle of the first course of PSMA-RLT the metastatic LN were measured smaller with computed tomography (CT) and the PSA level declined to 81.2 ng/mL (reduction of 85%). (**C**): One month after the third cycle of the second course of PSMA-RLT, these LN were either tiny or completely disappeared in the CT and the PSA level dropped further to 0.93 ng/mL (reduction of >95%). The overall survival of this patient was 263 weeks from the beginning of the first cycle of first course and 227 weeks from the beginning of the first cycle of second course of the PSMA-RLT.

The results of log-rank analyses to ascertain the overall survival of the patients by the type of metastasis indicated a significantly shorter survival of the patients who had metastatic bone lesions (M1b) compared with those with other types of metastases (M1a or M1c), they had a median survival of 123 weeks vs. not reached, *p* = 0.03, 95% CI 2.42–243, shown in Figure 3. Additionally, the existence of only lymph node metastases (M1a) was significantly associated with a longer survival compared with the availability of prostate metastatic lesions of other types (147 weeks vs. median survival not reached, *p* = 0.02). Figure 4 illustrates the ^68^Ga-PSMA-11 PET scan images of a patient with M1a demonstrated a highly favorable response to two courses of PSMA-RLT. 

While the results of the univariate analysis with the Cox regression model showed levels of Hb as well as serum alkaline phosphatase and PSA prior to the first cycle of the second PSMA-RLT course as predictors for OS in these retreated patients (all *p* < 0.05), in multivariate analysis, only the PSA levels remained significant for survival (*p* < 0.05, hazard ratio 2.43, 95% CI 1.01–5.87). Nevertheless, we did not observe a significant impact of other therapies, such as hormonal as well as chemo- and Ra-223 (Xofigo^®^) therapies, before the start of PSMA-RLT on the survival and PFS in our investigated cohort.

## 4. Discussion

Owing to the poor prognosis of the patients with mCRPC, and their low survival rate of less than 2 years from the time of their diagnosis, new therapeutic approaches and strategies are constantly striven to improve the survival and quality of life of these subjects [13]. 

In this study, we presented data of a rather selected mCRPC population, who previously benefited from PSMA-RLT and was retreated with another course consisting of three cycles of a standardized radionuclide therapy every 4 weeks, with a median interval of 16 weeks (range 4–96 weeks) between the first and the second therapy courses. Most of these patients were pretreated with enzalutamide and/or abiraterone as well as docetaxel and/or cabazitaxel therapy and the majority exhibited a good response rate and tolerability without having clinical and laboratory signs of severe therapy toxicities to the first PSMA-RLT course. 

Consistent with the outcomes of other previous studies where patients were retreated with PSMA-RLT [14,15], approximately 42% of the treated patients who responded to therapy showed a PSA reduction of greater than 50%. Indeed, the levels of PSMA expression in prostatic tumors, their related metastases and levels of serum PSA might not correlate with each other, as the expression of PSA, unlike PSMA, is mainly promoted by androgens and regulated by the androgen receptor [16]. In the prospective TheraP study by Hofman et al., PSA was used as the primary endpoint to evaluate the therapy response to both PSMA-RLT and cabazitaxel in mCRPC patients [8]. Consistently, response assessment based on PSA levels was one of the main endpoints of a single-center phase II prospective trial by Violet et al. [14], which included 50 mCRPC patients retreated with PSMA-RLT. In a study by Grubmüller et al., which involved patients who underwent the first PSMA-RLT course, treatment response was assessed in these mCRPC patients by comparing PSMA uptake in tumors and metastases before and one month after the third cycle of therapy using ^68^Ga-PSMA PET scan examinations. The results revealed a significant association of changes in the total tumor volume on the PSMA PET scan, but not in the RECIST (response criteria in solid tumors) evaluation with the PSA response [11]. Although it was not the focus of this current study, the PSMA PET parameters were a strong predictor of survival in men treated with PSMA-RLT in a study by Ferdinandus et al. [17]. 

In addition, the report of only two cases of grade 3 anemia and one case of severe thrombocytopenia suggests that the rate of treatment-related toxicity remained good and consistent with the results of previous studies [15,18,19]. These results, thus, indicated that a further therapy course with an additional three cycles of PSMA-RLT every 4 weeks is well tolerated and accompanied by satisfactory response rates to the treatment. Likewise, a second course of treatment for 30 mCRPC patients was also performed in a study by Yordanova et al. [20], and the outcomes have similarly demonstrated safety and efficacy of rechallenge PSMA-RLT. However, unlike our study, the first and second rechallenge therapy in that study were heterogenous regarding the injected activity (ranged 3.8–6.7 GBq) as well as the number of cycles in each course (ranged 1–6 cycles), and the interval between these cycles was ambiguous. In this respect, the diversity of the patients treated, and the differences in their tumor burden and comorbidities in our study and the study by Yordanova et al. should be taken into consideration. 

Furthermore, a median OS of approximately 4 years from the onset of the first cycle of the first course and a median OS of approximately 3 years from the date of initiation of the first cycle of the second therapy course were observed in our studied cohort. The median PFS with 31 weeks after the second therapy course was slightly superior to the median PFS from the last cycle of the first course until PSA progress (= 27 weeks). These findings are not unexpected given the growing recognition of the therapeutic efficacy and, thus, the prolonged survival of patients with advanced mCRPC who have acquired PSMA-RLT, as a PSA decrease of only ≥20% is predictive of prolonged survival [2,6,20]. The shortest OS was found in patients with M1c and the shortest time from the last treatment cycle to disease progression was identified in patients with M1b. Moreover, the patients who had only metastatic bone lesions lived significantly shorter than the patients with other types of metastases (median survival not reached vs. 123 weeks, *p* = 0.03). Although the type and distribution of metastases did not influence patient survival in the results of previous studies [10,12,21], the results of this current analysis indicated an association between the presence of lymph node metastases and longer survival, whereas the presence of bone metastases was significantly linked to a shorter survival in patients retreated with three cycles of PSMA-RLT every 4 weeks. In agreement with these findings, Ahmadzadehfar et al. have shown a negative impact of bone metastasis on the survival of patients treated with PSMA-RLT in 11 different clinics in a multicenter study, including data from more than 400 mCRPC patients [22]. Furthermore, the results of the same study showed 30 patients with only lymph node metastases that had the longest median OS among all other patients studied. In fact, at this advanced tumor stage with bone involvement, they often have diffused bone marrow metastasis, which limits the effectiveness of the therapy. Hence, patients with only lymph node metastasis might have a better outcome and a higher response rate to PSMA-RLT than patients with bone ± lymph node metastases. This has also been demonstrated in other studies [23,24], particularly in a study by von Eyben et al. in 45 patients with predominant lymph node metastatic prostate cancer [25]. 

Firstly, the retrospective design is the major limitation of the study. Secondly, the small sample of the included patients with different tumor burdens and diverse pretreatments will restrict the results of this investigation. However, this treated cohort represents the patient population referred to PSMA-RLT in clinical routine quite well. Additionally, although we have previously published part of our dosimetric data in a subgroup of patients who obtained the first PSMA-RLT course [26], the lack of such information in this current study could limit its outcomes. The reason lies in the crucial role of radiation dosimetry in estimating the therapy response and level of absorbed radiation dose, not only for each individual metastasis but also for the organs physiologically exhibiting an uptake of PSMA-RLT [27] and thereby evaluating their degree of therapy toxicity [28]. Moreover, no comparison has been conducted between PSMA-RLT and other hormonal or chemotherapies that might be optioned for these mCRPC patients at this stage of the tumor. Thus, the results of larger prospective studies such as VISION [29], comparing survival outcomes of patients receiving PSMA-RLT with those acquiring the best standard medical care, as well as the interesting results of the multicenter TheraP trial [8], should help to support the forthcoming implementation of this radionuclide therapy into the clinical treatment routine of patients with prostate cancer.

## 5. Conclusions

A second course of three cycles of standardized PSMA-RLT with only a 4-week interval between the cycles is safe and yields favorable tolerability, response rates, OS and PFS, thereby making it a promising option for the retreatment of mCRPC patients who have previously responded well to PSMA-RLT.

## Figures and Tables

**Table 1:** Clinical characteristics of the studied mCRPC patients prior to obtaining the second PSMA-RLT therapy.

Parameters	Values
Patients (*n*)	43
Age (mean ± SD) years	71.4 ± 6.6
Weight (mean ± SD) kilogram	83.1 ± 11.4
[^177^Lu]Lu-PSMA-617 MBq	7351 ± 647
≥50% PSA decline after 1st PSMA-RLT (*n*) %	(26) 60.5
Karnofsky score (*n*) %	
<80%	(16) 37.2
≥80%	(27) 62.8
ECOG index (*n*) %	
0	(8) 18.6
1	(26) 60.5
2	(9) 20.9
Previous treatments (*n*) %	
Enzalutamide/abiraterone	(27) 62.8
Docetaxel/cabazitaxel	(30) 69.7
Ra-223 (Xofigo^®^)	(12) 27.9
Metastatic lesions (*n*) %	
M1a	(8) 18.6
M1b	(27) 62.8
M1c	(8) 18.6

(*n*): number; MBq: megabecquerel; ECOG: Eastern Cooperative Oncology Group. M1a: lymph node only; M1b: bone ± lymph node without visceral metastasis; M1c: visceral metastasis.

**Table 2:** Comparison of laboratory parameters of the studied mCRPC patients before and after the second course of three cycles of PSMA-RLT every 4 weeks.

Parameters	Before Therapy	After Therapy	* q * -Value
* PSA µg/L	40.8 (0.87–1358)	20.2 (0.60–1962)	0.002
Hemoglobin g/dL (mean ± SD)	11.5 ± 1.7	11 ± 1.6	0.006
Thrombocyte g/L (mean ± SD)	208 ± 63	185 ± 63	0.002
* Leucocyte g/L	5.4 (1.17–14.3)	4.8 (2.1–14.1)	n.s.
* Creatinine mg/dL	0.96 (0.54–2.24)	0.94 (0.61–2.6)	n.s.
* Alkaline phosphatase U/L	78 (42–995)	84 (47–1345)	n.s.
* LDH U/L	205 (96–278)	194 (86–551)	n.s.

PSA: prostate-specific antigen; LDH: lactate dehydrogenase; n.s.: not significant; (*) data not normally distributed, presented in median and range and log_10_ transferred for analysis.

**Table 3:** Evaluation of treatment toxicities based on CTCAE version 4.0.

Parameters	Before Therapy			After Therapy		
	Toxicity (*n*)			Toxicity (*n*)		
	Grade 1	Grade 2	Grade 3	Grade 1	Grade 2	Grade 3
Hemoglobin g/dL	18	7	0	15	9	2
Thrombocyte g/L	8	0	0	8	0	1
Leucocyte g/L	2	0	1	3	1	0
Creatinine mg/dL	3	0	0	1	0	0

(*n*): number of reported cases.

**Table 4:** Median overall survival and median progression-free survival in weeks calculated from the time of the first and second PSMA-RLT course in relation to the type of metastases present.

Type of Metastasis	OS Calculated from 1st Course (Weeks)	OS Calculated from 2nd Course (Weeks)	PFS after 1st Course (Weeks)	PFS after 2nd Course (Weeks)
Total population	188	136	27	31
M1a	>169 *	>147 *	41	32
M1b	176	123	25	24
M1c	119	106	44	40

M1a: patients with lymph node metastasis; M1b: patients with bone ± lymph node without visceral metastasis; M1c: patients with visceral metastasis; OS: overall survival; PFS: progression-free survival; (*): no patient died in this group during follow-up.

## Data Availability

Not applicable.
